# Cattle Welfare in Dairy and Beef Systems: A New Approach to Global Issues

**DOI:** 10.1017/awf.2024.17

**Published:** 2024-04-17

**Authors:** John Webster

**Affiliations:** University of Bristol, Bristol

This volume comes as a successor to *The Welfare of Cattle* (2008) and reviews recent advances in animal welfare science within four main themes:

1. Behavioural, physiological and immunological indices of ‘a good life’.
2. Challenges and solutions in extensive and intensive management systems.
3. Cattle welfare in different contexts: at calving during growth and at slaughter. Precision livestock farming, strategies and tools for genetic selection.
4. Cattle welfare: culture and sustainability interactions, smallholder farming in sub-Saharan Africa, challenges of ‘new entry’ dairy farming.

The various authors of the individual chapters have been appropriately selected for their scientific and practical experience and the overall result is a set of comprehensive reviews of important issues. The topics addressed in the individual chapters cover a very wide range, from new science in genomics and molecular biology to the eternal practical challenges and problems faced, for example, by subsistence farmers in sub-Saharan Africa and new entries to dairy farming, whether intensive or extensive.

There is a lot of good stuff in this book. Nevertheless, I suggest that I might not have been the right person to invite for a review, as I come to this after over 60 years of study and action with regard to cattle physiology, health and welfare and, amongst some good new stuff, I am fed concepts and ideas that I have read and witnessed many, many times before. Moreover, about 80% of the references relate to papers published in the last twenty years. I acknowledge that this may be appropriate for a volume that reviews advances since its predecessor was published in 2006. I recognise also that each new scientist starts from scratch. However, perhaps the strongest message that keen young scientists can draw from these comprehensive lists of references is to highlight areas where our knowledge is now comprehensive and suggest (indirectly) new areas of study that would be more interesting, and more useful.

Given the sheer breadth covered, allowing individual chapter purchase would be a bonus, especially if the cost of the book is prohibitive for certain students. The good news is that I am advised by the senior editor of this series, Professor Clive Phillips, that students from certain low-income areas will be able to download individual chapters free of charge. Young welfare scientists and others keen to advance their understanding of cattle husbandry, health and welfare may derive much of value from individual chapters that cater for their individual needs. The science-oriented chapters are also comprehensively equipped with enough references to bulk out any PhD thesis.

